# Editorial: Parents with mental and/or substance use disorders and their children, volume III

**DOI:** 10.3389/fpsyt.2025.1630833

**Published:** 2025-06-05

**Authors:** Joanne Nicholson, Jean Lillian Paul, Geneviève Piché, Anja Wittkowski

**Affiliations:** ^1^ The Heller School for Social Policy and Management, Institute for Behavioral Health, Brandeis University, Waltham, MA, United States; ^2^ Department of Psychiatry, Psychotherapy, Psychosomatics, and Medical Psychology, Division of Psychiatry I, Medical University Innsbruck, Innsbruck, Austria; ^3^ Département de Psychoéducation et de Psychologie, Université du Québec en Outaouais, Saint-Jérôme, QC, Canada; ^4^ Division of Psychology and Mental Health, School of Health Sciences, Faculty of Biology, Medicine and Health, The University of Manchester, Manchester, United Kingdom; ^5^ Perinatal Mental Health and Parenting (PRIME) Research Unit, Department of Research and Innovation, Greater Manchester Mental Health National Health Service (NHS) Foundation Trust, Manchester, United Kingdom

**Keywords:** parents, mental health, substance use disorders, children, families, family-focused practice

## 1 Introduction

This is the third Frontiers Research Topic volume focusing on parents with mental and/or substance use disorders and their children. Once again, international researchers actively contributed, with 33 articles written by 179 authors from 13 countries, including Austria, Australia, Canada, the Czech Republic, Germany, Ireland and Northern Ireland, the Netherlands, Norway, Sweden, Switzerland, the United Kingdom, and the United States. Earlier volumes (1, 2) focused on the experiences of families living with parental mental illness, emphasizing the need for rigorous research designs, innovative methods, and the development and testing of interventions for adults, children, and their families. These prior volumes reflected a gradual shift in the work, from exploring experiences and documenting prevalence, to developing solutions for supporting family members in optimizing outcomes.

In announcing Volume III, we acknowledged the need for widespread knowledge translation and dissemination efforts, with an eye towards improving both policy and practice. Our goal was to engage international colleagues in highlighting the nuanced needs of diverse target populations, treatment settings, and service contexts; elevating policy issues impacting these families; and further developing and testing interventions to build the evidence base. The submitted articles illustrate updates in our knowledge regarding the prevalence and characteristics of adults and children living with parental mental illness. The experiences and needs of mothers and families in the perinatal period are elaborated on and addressed in conceptualizing intervention opportunities, targets and outcomes. Innovative measures and methods are highlighted, along with the development, adaptation, implementation, and testing of family-focused interventions. Workforce efforts and challenges in sustaining interventions are described.

## 2 Describing adults and children when there is parental mental illness

Nine papers in this Research Topic focus on the characteristics of children or adults when there is parental mental illness in the family. These papers include one systematic review (Schoors et al.), seven original research articles (Bérubé et al., Davidson et al., Kinzenbach et al., Luczejko et al., Nevard et al., Seipp et al., Werkmann et al.) and one perspectives piece (Allchin et al.). Study designs are all cross-sectional and include six quantitative studies (Bérubé et al., Davidson et al., Luczejko et al., Seipp et al., Werkmann et al., Isobel), one using mixed methodologies (Kinzenbach et al.) and one qualitative study (Nevard et al.). Articles reflect work in Germany, Canada, United Kingdom, Belgium, and Australia. Four are from the large COMPARE trial (Kinzenbach et al., Luczejko et al., Seipp et al., Werkmann et al.), the protocol of which was published in the second volume of this Research Topic (3).

Following their studies, these authors suggest target areas for new interventions to support family mental health. They argue for the involvement of physical activity in family-focused interventions, both for parents and children, with the knowledge that physical and mental health are interconnected (Davidson et al.). For parents, they propose including a specific focus on parental stress (Seipp et al.), and emotional regulation (Bérubé et al., Luczejko et al.) to enhance parenting skills. For children, in addition to physical activity, they suggest that interventions cover facial emotional recognition, which could be impaired in children of parents with a mental illness (Werkmann et al.), and helping children build their social networks, recognizing the active role children have in navigating the social and formal relationships around them (Nevard et al.). Overall, the need for longitudinal studies and a consideration of the developmental stage of the child during the parent’s acute phase of mental illness is highlighted. Finally, following the sustained growth of research and practice in family mental health studies, one paper calls for a reflection on the “frame” of risk and vulnerability and its potential impacts on families and services (Allchin et al.).

## 3 Focusing on the perinatal period

A number of papers in this volume bring attention to the experiences and needs of mothers (and fathers) during the perinatal period, particularly related to their mental health and the parenting of infants (Isobel, Hölzle et al., O’Brien et al., Reid et al., Millard and Wittkowski, Wozniak et al., Shone et al., Schöch et al.). The papers are largely exploratory, engaging women and professionals who highlight the importance of positive connections (i.e., with therapist, baby and motherhood), elaborate important mechanisms for change (e.g., sensitivity and engagement in the therapeutic relationship), and set priorities for future interventions (O’Brien et al., Reid et al.).

Wozniak et al. focus on changes in maternal *self-construal* following admission to Mother and Baby units in England, assessing how mothers view themselves in relation to self and others. Most mothers report positive and more adaptive changes to self-esteem and self-perception. Mothers referred to a Mother Baby unit in Australia provide evidence of significant rates of lifetime trauma exposure, with 24% meeting criteria for complex PTSD (Isobel), suggesting the importance of attending to the ways in which trauma histories are likely to impact mothers’ relationships with infants. Isobel suggests the potential benefits of a shift in perinatal mental health treatment to include a focus on navigating the transition to parenting and considering the impact of trauma in transgenerational attachment.

Millard and Wittkowski reviewed the current literature on compassion focused therapy (CFT) offered to women who are mothers in the perinatal period. The authors underscore the limited evidence-base and the need for future research. Eleven perinatal mental health peer support programs were identified in a systematic review of the literature (14). Authors highlight the key considerations in perinatal peer support, including contextual background, provider training and support, delivery modes and locations and, ultimately, evaluation. Sharing lived experience and providing individualized, flexible support were central to all identified peer support programs. The authors recommend future research to specify and track action mechanisms, and to generate practical information regarding program implementation and impact in addition to effectiveness metrics. As suggested in other papers in this volume, the authors emphasize the benefit of diverse stakeholder engagement in the evaluation of services to include individuals receiving services, providers, and community members. Shone et al. conducted a systematic review of clinician rated measures to assess the parent-infant relationship. They found three instruments to have the most promising results in terms of overall quality (including: Mother-Infant/Toddler Feeding Scale, Tuned-In Parenting Scale, and Coding Interactive Behavior instrument). However, they concluded that measures had low methodological and psychometric evidence and argue for further research to determine most suitable assessment measures.

Recommendations across papers for future efforts in perinatal intervention development and testing include the importance of understanding mothers’ experiences and needs, tailoring interventions to be individualized and flexible, specifying action mechanisms, and building an evidence base, actively informed by professionals, researchers, and mothers themselves.

## 4 Developing measures and methods in family mental health research

Seven papers in this special volume introduce new measures or methods that advance parental mental health research (Schöch et al., Fahrer et al., Piché et al., Stracke et al., Reupert et al.). These contributions come from diverse international contexts, including Germany (Fahrer et al., Stracke et al.), Austria (Schöch et al.), Australia (Reupert et al.), and an international collaboration with the leading author based in Canada (Piché et al.). Two of these papers again are part of the large COMPARE project (Fahrer et al., Stracke et al.), which continues to make substantial contributions to the field.

Among these studies, Fahrer et al. present a new method for measuring interactive behaviors between parents and children when a parent has a mental illness, offering valuable insights into family dynamics that can inform intervention development. Stracke et al. contribute by validating and shortening a stigma questionnaire specifically designed for children of parents with a mental illness, making it more accessible and efficient for both research and clinical settings (Stracke et al.). Meanwhile, Schöch et al. critically review existing screening tools for paternal perinatal mental illness, identifying gaps and proposing refinements to enhance their accuracy and relevance. Notably, they highlight that the widely used Edinburgh Postnatal Depression Scale (EPDS) may fail to capture atypical depressive symptoms in men, such as irritability, aggression, or substance misuse.

The scoping review by Piché et al. highlights significant gaps in qualitative research on parental mental illness, particularly the need to capture diverse family experiences—such as those of children, fathers, and other family members. The authors emphasize the need for holistic research approaches to better understand how parental mental illness impacts family systems. The authors also advocate for greater family involvement in setting research priorities to enhance the quality and relevance of future work. Lastly, Reupert et al. apply the Social Return on Investment (SROI) approach to evaluate a program for children and young people who have families living with mental illness, highlighting the broader social value of such interventions.

Together, these contributions exemplify how rigorous methodological innovation can expand our capacity to measure, understand, report and respond to the complex challenges faced by families experiencing parental mental illness.

## 5 Advancing family-focused interventions: insights and directions

This volume highlights the critical role of family-focused interventions in addressing the needs of children and families facing parental mental illness. Furlong et al. present findings from a randomized controlled trial conducted in Ireland considering program cost as well as family outcomes, building on two qualitative studies published in Volume II (2). Their results underscore the positive effects of the Family Talk program in improving family functioning, child behavior, and mental health literacy. The authors suggest consideration of the impact of family context on intervention outcomes Families with stronger partner and socioeconomic supports experienced greater benefits, particularly in reducing child anxiety and depression, suggesting that contextual factors can significantly shape intervention effectiveness. These results underscore the importance of developing a continuum of services, ranging from lower to higher intensity interventions, to meet the diverse needs of families adequately.

Four papers focus on novel family approaches to care, integrating services for adults and children, across agencies and services. Stolper et al. offer a family case study of an integrated family approach in mental health care, with treatment being offered by professionals in adult and child mental health services in the Netherlands. This paper describes their approach in detail by highlighting how service providers could liaise with each other about the family and what domains they would need to consider alongside the family in its environment. The authors identify barriers to keeping the whole family in mind, including organizational policy, interagency collaboration, professionals, and patients themselves. Stolper et al. further demonstrate how such interventions enhance the quality of parent-child interactions, parental sensitivity, and reflective functioning in a study with a mixed methods design, using questionnaires, an observation instrument, and semi-structured interviews.

Can et al. point to the lack of research linking the treatment of severe parental mental illnesses, such as bipolar disorder and schizophrenia, to child outcomes. They call for future studies to integrate intergenerational outcomes into adult-focused trials, advancing our understanding of how to support families impacted by severe parental mental illness. Dunn et al. emphasize the critical role of co-design in the development of parenting interventions by outlining a collaborative intervention development project. The investigators demonstrate the value of actively engaging patients, caregivers, and professionals in the intervention design process to ensure that programs are not only relevant and acceptable, but also practical and sustainable in real-world contexts.

Intervention sustainability is another key theme in this volume. Allchin and Albermann et al. identify barriers to sustaining family-focused interventions, including insufficient training for practitioners, a lack of organizational support to identify clients’ parental roles, and systemic reliance on biomedical, individual-focused models of care. The authors underscore the importance of collaborative decision-making between practitioners and families, and involving families in evaluating the effectiveness of interventions, converging with conclusions from Dunn et al. They also recognize the diversity of family needs, aligning with Furlong et al.

Collectively, these papers advocate for a shift toward family-focused, participatory, and sustainable practices in mental healthcare to enhance outcomes for families. They emphasize the critical need for interdisciplinary collaboration, recognizing that families with parental mental illness have complex family needs. The authors call for sustained investment in evidence-based interventions that are not only scalable but also adaptable to diverse cultural, socioeconomic, and resource-limited settings. By championing approaches that prioritize inclusivity, co-design with stakeholders, and long-term sustainability, these studies underscore the importance of reimagining mental healthcare systems to support families with parental mental illness better, across generations.

## 6 Addressing workforce challenges

Understanding the challenges that health and social care professionals face in their efforts to support the mental health and wellbeing of parents and their children is critical to the successful implementation of interventions and family-focused approaches. The views and experiences of professionals in different service and research contexts and in different countries are explored in several papers.

Zegwaard et al. describe the implementation of innovative interagency, family-focused case consultation teams, which boosted practitioner confidence in supporting families with complex challenges, six months post-implementation. Their study underscores the need for sufficient time, resources, and expert support to enhance interagency collaboration and address complex family needs. In a study of the predictors of family-focused practice, professionals working with adult mental health patients in Quebec, Canada, completed the Family Focused Mental Health Practice Questionnaire (Piché et al.). Apart from working full-time, the strongest predictors for the adoption of higher family-focused practice levels among these professionals are, similar to the findings of Zegwaard et al., having a perceived higher level of skills, knowledge, and confidence towards FFP and having a supportive workplace environment.

Oakes et al investigated the views and experiences of health and social care professionals recruited from six National Health Service (NHS) and Local Authority settings in England to gain a better understanding of their experiences supporting parents with serious mental illness (e.g., schizophrenia and bipolar disorder) and their children. Professionals indicate that available services remain inaccessible and unacceptable to these parents. They further describe experiencing a conflict in their attempts to balance the needs of parents with those of their children. These authors highlight the need for specialist family-focused services, with collaboration across health and social care settings. The professionals in their study report the need for greater service knowledge and more training in parenting with mental illness, which supports Piché et al.‘s findings.

As part of the implementation of the ParentingWell Practice Approach, adapted from Let’s Talk about Children, practitioners working with adults with mental illness were invited to participate in the ParentingWell Learning Collaborative, including in-person orientation and learning sessions, and virtual follow-up coaching sessions (Heyman et al.). Participants report that coaching sessions allowed practitioners to share concrete approaches to supporting parents, and to reflect more deeply on the needs of these parents alongside their own personal experiences and need for self-care in this field of work.

Linderborg et al. explored the utility of the Family Model in Swedish child/adolescent and adult mental health services with clinicians and managers in a qualitative study using naturalistic enquiry. Their analyses on a meta understanding level indicate that the Family Model was perceived as a useful tool for families, with its potential to influence prevention and to bridge the gap between child and adult mental health services. The Family Model seemed to empower families with its focus on strengths, which in turn motivated clinicians to use it more. The authors recommend the gradual implementation of this model following training and ongoing guidance in its use, comparable to Piché et al.‘s findings. Sufficient time and resource allocations alongside family “champions” in organizations are also recommended.

Given the well-documented barriers (e.g., organizational policy, interagency collaboration, professional and parental factors) to keeping the whole family in mind or to implementing a family-focused practice, Stolper et al. offer the main elements for such an approach to succeed, based on their work with professionals and parents. Once more, the need for integrated, multidisciplinary and multiagency work, offered by a specialist team, is stressed.

## 7 Next steps

The contributions in this volume highlight significant advancements in understanding and addressing the needs of families when a parent has a mental illness. However, they also point to critical gaps in research, intervention development, and systemic integration that require further attention. In this discussion, we outline key directions for future efforts across intervention development, research priorities, workforce considerations, and broader systemic and policy implications.

### 7.1 Rethinking mental health systems: supporting families across the lifespan

One overarching theme emerging from this volume is the need to rethink mental health care systems to accommodate the needs of diverse families better at different life stages. The articles highlight the necessity of a continuum of services, ranging from early prevention efforts in the perinatal period to long-term support for families with older children. The development of new interventions must be informed by a nuanced understanding of the characteristics and needs of parents, children, and families, emphasizing the following aspects.

#### 7.1.1 An integrated family approach

Future interventions should move beyond individual-focused treatments to incorporate family dynamics, considering how mental illness impacts relationships within the household and/or the wider family. This approach includes a greater emphasis on the roles of fathers, non-birthing parents, and other caregivers beyond the traditional focus on mothers, acknowledging the diverse ways in which families function and provide support.

#### 7.1.2 Trauma and transgenerational attachment

Recognizing the role of trauma in shaping parent-child interactions is critical. Interventions should address how past experiences influence present parenting behaviors and attachment patterns. Additionally, interventions should actively include the perspective of the developing child. For example, considering the infant’s early communicative signals and relational needs may be central to intervention strategies.

#### 7.1.3 Specification and tracking of action mechanisms

Research must clearly define and measure the mechanisms driving intervention effectiveness, ensuring that programs are replicable and adaptable across diverse populations. We should reconsider levels of evidence to inform policy and advocate for a broader understanding of the ways in which research can support and guide practice change. This approach includes expanding definitions of what constitutes valid and impactful evidence, integrating qualitative insights, and recognizing the value of participatory and co-designed approaches in shaping effective interventions.

#### 7.1.4 Contextual factors shaping intervention outcomes

Family structure, socioeconomic status, and access to resources are significantly related to the effectiveness of interventions. Future work should consider these factors in program design and evaluation. Particular attention should be paid to the experiences of non-traditional family structures, including single-parent households, same-sex parents, and extended kinship care arrangements, ensuring that interventions are inclusive and relevant across diverse caregiving contexts. We should also consider the family and social impact on these broader caregivers in providing unpaid social support.

#### 7.1.5 Sustainability and adaptability

Programs should be designed for long-term implementation, considering workforce training, resource allocation, and cross-sector collaboration to maintain impact over time.

### 7.2 Advancing research: Methodological considerations and emerging priorities

Future research must build on existing knowledge while addressing current methodological limitations. Several key research directions emerge.

#### 7.2.1 Mixed methods approach

Integrating qualitative and quantitative methods can provide a richer understanding of family experiences, intervention processes and, ultimately, intervention effectiveness.

#### 7.2.2 Longitudinal research

More studies following families over time are needed to understand the long-term effects of parental mental illness and interventions designed to support them. We should advocate for the importance of such approaches with funders, universities, and our government partners.

#### 7.2.3 Cross-sector and intergenerational research

Collaboration across health, education, legal, and social services is essential to developing a holistic understanding of family needs.

#### 7.2.4 Innovating measurement strategies

Identifying new ways to assess program effectiveness, including culturally relevant tools for measuring family-focused practice, family relationships, child well-being, and service engagement, will strengthen the evidence base.

#### 7.2.5 Co-design and participatory research

Involving parents, children, and service providers in research design and intervention evaluation ensures that studies are relevant, feasible, and aligned with the needs of those they aim to support. Efforts should also be made to include infants’ experiences by developing methods that capture their relational and emotional worlds in ethical and developmentally appropriate ways.

#### 7.2.6 Enhancing diversity in research participation

Studies must include a broader range of family structures, cultural backgrounds, and lived experiences to ensure findings are generalizable and inclusive.

### 7.3 Workforce development: building capacity for family-focused practice

Effective implementation of family-focused interventions relies on a well-trained, confident, and supported workforce. The findings in this volume underscore several priorities for workforce development.

#### 7.3.1 Well-specified training approaches

Training for mental health and social service providers should be structured, evidence-based, and tailored to the realities of family-focused practice, to build expertise in working with families.

#### 7.3.2 Organizational support and resource allocation

Institutions must provide sufficient time, funding, and systemic backing to ensure family-focused approaches are sustainable.

#### 7.3.3 Championing organizational change

Identifying and supporting leaders within organizations who advocate for family-focused care can facilitate widespread adoption and integration.

#### 7.3.4 Embedding reflective practice

Encouraging practitioners to engage in self-reflection enhances their ability to navigate complex family dynamics and improve service delivery. This practice includes reflection on our biases and gendered assumptions in caregiving, and ensuring that professionals are attuned to the perspectives and voices of all family members, including fathers, infants, and all caregivers.

### 7.4 Addressing underexplored areas and broader policy implications

While this volume covers a wide array of topics, certain areas remain underexplored and warrant further investigation.

#### 7.4.1 Fathers’ experiences and roles

Much of the research to date and reported in our Research Topics has focused on mothers, leaving a gap in understanding how paternal mental health influences family dynamics and child outcomes. Future work must examine the specific challenges fathers face, the support they require, and how interventions can engage them better in family-focused care.

#### 7.4.2 Children’s participation in research

Ethical and methodological considerations must be addressed to involve children meaningfully in research while safeguarding their well-being.

#### 7.4.3 Cultural norms and parenting expectations

Understanding how cultural beliefs shape perceptions of mental health and parenting can inform the design of more culturally responsive measures, methods, and interventions.

#### 7.4.4 Substance use disorder and other co-occurring conditions

Exploring how parental substance use interacts with mental health challenges and impacts family well-being continues to be a critical area for study. Other medical conditions may co-occur with or convey mental health challenges, with exacerbated impact on families as well.

#### 7.4.5 Parents with disabilities

Research should consider the unique needs and strengths of parents with disabilities in the context of accessible mental health support.

#### 7.4.6 Policy impact and implications

Policymakers must be engaged in translating research findings into actionable strategies that improve service access, funding, and regulatory frameworks. This approach includes recognizing the importance of inclusive family policies that consider the diversity of caregiving roles and family leave, and ensure that fathers, extended family members, and non-traditional caregivers are supported within mental health systems.

## 8 Conclusion

This volume illustrates the growing momentum in research and practice aimed at supporting families in which parents have a mental and/or substance use disorder. As the field continues to evolve, a commitment to interdisciplinary collaboration, participatory research, and evidence-informed practice is essential. By addressing gaps in intervention development, research methodology, workforce training, and systemic integration, we can move towards a more comprehensive and sustainable approach to family mental health care and wellbeing. The challenge now is to translate these insights into action—ensuring that families receive the support they need, not only in crisis but throughout the course of their lives.

We would like to express our heartfelt appreciation to Dr. Tytti Solantaus and Dr. William Beardslee, whose pioneering work has significantly shaped the field of family mental health. Their dedication to understanding and supporting families affected by parental mental illness has not only advanced scientific knowledge, but also transformed clinical practice and policy worldwide. Dr. Solantaus’s contributions have emphasized the strengths and resilience of families, inspiring innovative, preventive approaches that acknowledge the experiences of both parents and children. Dr. Beardslee’s groundbreaking work on resilience and family-focused interventions laid the foundation for evidence-based programs that continue to guide researchers and practitioners in their efforts to improve outcomes for families. Their wisdom, compassion, and commitment have shaped a field that is deeply attuned to the needs of families. We are honored to build upon the foundation they have laid.
